# Spectrum of Infection and Risk Factors for Human Monkeypox, United States, 2003

**DOI:** 10.3201/eid1309.070175

**Published:** 2007-09

**Authors:** Mary G. Reynolds, Whitni B. Davidson, Aaron T. Curns, Craig S. Conover, Gregory Huhn, Jeffrey P. Davis, Mark Wegner, Donita R. Croft, Alexandra Newman, Nkolika N. Obiesie, Gail R. Hansen, Patrick L. Hays, Pamela Pontones, Brad Beard, Robert Teclaw, James F. Howell, Zachary Braden, Robert C. Holman, Kevin L. Karem, Inger K. Damon

**Affiliations:** * Centers for Disease Control and Prevention, Atlanta, Georgia, USA; † Illinois Department of Public Health, Chicago, Illinois, USA; ‡ Rush University, Chicago, Illinois, USA; § Wisconsin Department of Health and Family Services, Madison, Wisconsin, USA; ¶ Kansas Department of Health and Environment, Topeka, Kansas, USA; # Indiana State Department of Health, Indianapolis, Indiana, USA

**Keywords:** Monkeypox, epidemiology, risk factor, smallpox vaccination, zoonotic, subclinical infection, research

## Abstract

For the 2003 monkeypox virus (MPXV*)* outbreak in the United States, interhuman transmission was not documented and all case-patients were near or handled MPXV-infected prairie dogs. We initiated a case–control study to evaluate risk factors for animal-to-human MPXV transmission. Participants completed a questionnaire requesting exposure, clinical, and demographic information. Serum samples were obtained for analysis of immunoglobulin G (IgG) and IgM to orthopoxvirus. When data were adjusted for smallpox vaccination, case-patients were more likely than controls to have had daily exposure to a sick animal (odds ratio [OR] 4.0, 95% confidence interval [CI] 1.2–13.4), cleaned cages and bedding of a sick animal (OR 5.3, 95% CI 1.4–20.7), or touched a sick animal (OR 4.0, 95% CI 1.2–13.4). These findings demonstrate that human MPXV infection is associated with handling of MPXV-infected animals and suggest that exposure to excretions and secretions of infected animals can result in infection.

## Abstract 2

Infection is associated with proximity to virus-infected animals and their excretions and secretions.

Monkeypox (MPX) is a smallpox-like, but zoonotic, disease endemic to regions of West and Central Africa. This disease results from infection with *Monkeypox virus* (MPXV) (*1*), a member of the genus *Orthopoxvirus* within the family *Poxviridae* (*2*). The principal animal reservoir of MPXV is unknown, but varied sylvan species are susceptible to MPXV infection (*3*–*6*), and multiple species have been implicated in virus transmission to humans (*7*,*8*). Identification of specific human activities (e.g., animal trapping, hunting, or skinning) or types of exposure to animals (e.g., bites; exposure to feces, urine, or respiratory droplets) that could result in MPXV transmission from animals to humans has been difficult to study because of the remote geographic locations involved and the retrospective nature of case reporting and investigation.

The introduction of MPXV into the United States in 2003 in a consignment of wild-caught animals (exotic pets) from Ghana led to the occurrence and recognition of the first human infections outside Africa (*8*). Although MPXV can be transmitted from person to person, no instances of interhuman transmission were documented during the US outbreak (*3*,*9*), which ultimately resulted in 47 confirmed and probable human cases (*10*). Manifestations of human illness seen during this outbreak were considered to have been mild relative to those observed for persons infected with Central African variants of the virus (*11*). All human case-patients were documented to have handled or to have been near infected prairie dogs that had been temporarily housed in the same complex as MPXV-infected, imported rodents. (One case-patient recalled no specific exposure but had ample opportunity for exposure.) The infected prairie dogs were sold as pets, and most ended up in private homes. In this environment, persons had diverse opportunities for exposure to infected animals, varying from being in the same room with an infected animal to cleaning the cage of an infected animal. In addition, several animals were transported to veterinary clinics for evaluation after they became ill, which increased the potential range of human exposures to infected animals. The US outbreak provides a means to identify activities or types of direct or indirect exposure to infected animals that may have been associated with increased risk for MPXV transmission from animals to humans.

A case–control study was conducted to evaluate risk factors for infection among persons exposed to MPXV-infected prairie dogs during the US outbreak in 2003. This study constituted a single arm of a multiobjective investigation exploring questions related to development of immune responses, infection sequelae, and the relationship between route of infection to MPXV and clinical outcomes. The purposes of this study were to identify independent risk factors associated with MPXV infection and disease in humans and to examine the role of previous smallpox vaccination and age in shaping human susceptibility to MPXV infection.

## Patients and Methods

### Human Study Participants

The protocol for this study (part of the multi-objective study) was reviewed and approved by the institutional review board for human subjects research at the Centers for Disease Control and Prevention (CDC) (Atlanta, GA, USA). Approval was received (by deferral) from partner institutions in Wisconsin (Wisconsin Department of Health and Family Services), Illinois (Illinois Department of Public Health), Indiana (Indiana State Department of Health), Kansas (Department of Health and Environment), and Georgia (Emory University). No monetary incentives or other forms of compensation were provided to study participants.

### Definitions and Participant Enrollment

The 47 persons who met criteria for confirmed or probable MPXV infection on the basis of a combination of clinical symptoms, exposure information, and laboratory criteria (*12**–**14*) (www.cdc.gov/ncidod/monkeypox/casedefinition.htm), and who had exposure to an infected prairie dog, were considered eligible for enrollment as study case-patients. Standardized case definitions were used to assign confirmed and probable case status (Table 1). Persons defined as having probable cases were those who had illness onset <21 days of exposure to MPXV who experienced fever (>37.4°C) and vesicular pustular rash, or rash (potentially uncharacterized) plus had immunoglobulin (IgM) to orthopoxvirus. MPX cases were confirmed on the basis of any of the following laboratory findings from clinically derived specimens: MPXV isolation, detection of MPXV-specific nucleic acid signatures, positive electron microscopy findings, or positive immunohistochemical findings (the last 2 in the absence of other orthopoxvirus virus exposures).

**Table 1:** Criteria used to define categories of study participants, monkeypox virus outbreak, United States, 2003*

Study classification	Classification by case definition†	Criteria met		
		Epidemiologic‡	Clinical§	Laboratory¶
Case	Confirmed	Yes	Yes	Yes#
	Probable	Yes	Yes (fever with vesicular pustular rash, or rash of unspecified type plus IgM)	No (if rash type unspecified, IgM 7–56 d after rash onset)
Not included	Suspect	Yes	No (fever or rash of unspecified type)	No
Control**	Not a case	Yes	No	No
Infected but not diseased	Unclassified	Yes	No	No (IgM detected at time of study)

*IgM, immunoglobulin M. †Available from www.cdc.gov/ncidod/monkeypox/casedefinition.htm, January 2004 (*3*,*12*,*13*). ‡Epidemiologic criteria for classification of monkeypox cases included exposure to an exotic or wild mammalian pet (obtained on or after the known importation event) exhibiting signs of illness (e.g., conjunctivitis, respiratory symptoms, and rash); exposure to an exotic or wild mammalian pet that had been exposed to an animal infected with monkeypox; or exposure to a suspected, probable, or confirmed human case of monkeypox. §Clinical criteria were rash (macular, papular, vesicular, or pustular; generalized or localized; discrete or confluent) plus fever (subjective or measured temperature >99.3°F [>37.4°C]), plus >2 other signs and symptoms (chills and/or sweats, headache, backache, lymphadenopathy, sore throat, cough, shortness of breath), all beginning <21 d after last possible exposure. ¶Laboratory criteria included culture of monkeypox virus, or demonstration of monkeypox virus DNA by PCR from patient clinical specimens, or demonstration of virus morphologically consistent with an orthopoxvirus by electron microscopy or immunohistochemical testing methods (in the absence of exposure to another orthopoxvirus). #Positive laboratory findings were sufficient to confirm a monkeypox case in the absence of complete clinical or epidemiologic history. **Persons investigated but ruled out as having monkeypox virus infections were eligible to enroll in the study as controls; additional controls were identified as in Patients and Methods.

Persons eligible for enrollment as study controls were those who 1) had exposure to an infected prairie dog either in a household or workplace setting, 2) did not acquire MPX disease (i.e., did not meet the clinical criteria for MPX disease as outlined in the case definition), and 3) did not show signs of infection in the absence of disease (i.e., were negative for IgM to orthopoxvirus).

Local health department personnel contacted potential study case-patients by telephone to ascertain their willingness to participate in the study and to request the names of potential family or social contacts. Persons who volunteered were then enrolled in person at the time of interview, and written consent was obtained. Thirty case-patients and 35 potential controls ultimately consented to participate in the study.

Three persons enrolled as potential controls were found to have elevated levels of IgM to orthopoxvirus. These persons were excluded as controls and were classified as MPXV infected without clinical disease. These 3 infected but not diseased case-patients were retained in a separate category to allow assessment of MPXV infection, as well as MPX disease, as an outcome measure.

### MPXV-infected Prairie Dogs

A prairie dog was defined as being MPXV infected if tissues obtained postmortem were positive for MPXV DNA by culture or PCR (*15*). Alternatively, in the absence of laboratory testing, the animal was defined as being infected if it had signs and symptoms of MPXV infection and was housed at a facility where other animals known to have been MPXV infected were also kept (*5*,*16*).

### Data Collection

Beginning in late July 2003 (≈40 days after illness onset of the last known human MPX case), face-to-face interviews were conducted with case-patients and controls. A standardized questionnaire was used to obtain information regarding demographic profile, exposure to potentially infected animals and infected humans, signs and symptoms of illness, and factors that might affect susceptibility, including smallpox vaccination history. The data used in this study pertaining to type of animal exposure have been summarized (*10*).

### Blood Collection and Serologic Analysis

At the time of interview, a blood sample was requested from all study participants for detection of antibodies to orthopoxvirus and investigation of cellular immunity. Donation of a blood specimen was not required for enrollment in this study, but those specimens that were obtained were assayed for IgG and IgM reactive with orthopoxvirus antigen (vaccinia) according to the methods of Karem et al. (*12*).

### Data Analysis

Demographic and medical history data were analyzed as dichotomous categorical variables (i.e., present vs. absent). Two classification schemes were used for age groups; one grouped participants according to whether they were adults (>18 years of age) or children (<18 years of age) and the other grouped participants according to whether they would have had an opportunity to have received smallpox vaccine as a routine childhood immunization (>33 and <33 years of age, respectively). Routine smallpox vaccinations stopped in the United States in 1972. Regarding the setting in which the exposure occurred, if a participant indicated he or she was exposed at home and an additional setting, home was noted as the principle setting, otherwise the response was left as recorded. Occupation was stratified into nonanimal-related versus animal-related. Animal-related occupations were veterinarian, veterinarian technician or assistant, or pet store employee. Responses from participants who indicated exposure to >1 infected prairie dog were combined into 1 response; affirmative responses were selected over negative responses when exposure to different animals was not uniform.

Odds ratio (OR) with 95% confidence interval (CI) was calculated to examine associations between disease status and exposure variables (e.g., age and smallpox vaccination status). Multiple logistic regression analysis was used to obtain ORs adjusted for the effect of smallpox vaccination (*17*) and to examine the risk for disease or infection associated with given exposures. The level of statistical significance established for the analysis was p<0.05. The small size of the study population precluded further assessment of independent risk factors. All statistical analyses were performed by using SAS version 9.1 (SAS Institute, Cary, NC, USA).

## Results

### Characteristics of Case-Patients and Controls

Sixty-one persons from 4 states affected during the MPX outbreak were enrolled in the study (Table 2). The 2 categories of MPX case-patients evaluated were persons who met the epidemiologic case definition for confirmed or probable MPX (case-patients, n = 30) and persons who did not have illness meeting the case inclusion criteria but who had elevated levels of IgM to orthopoxvirus at the time of enrollment and sampling (infected but not diseased [IBND] patients, n = 3). Twenty-eight study controls were identified from the pool of enrollees. All case-patients and controls had exposure to ill or infected prairie dogs; 4 persons reported exposure to other potentially infected animals (i.e., African rodents).

**Table 2:** Characteristics of monkeypox case-patients and controls selected from the population of persons exposed, monkeypox virus outbreak, United States, 2003

Characteristic	Study classifications, no. (%)		
	Case-patients	Case-patients infected but not diseased	Controls
State			
Illinois	8 (26.7)	1 (33.3)	8 (28.6)
Indiana	10 (33.3)	1 (33.3)	17 (60.7)
Kansas	1 (3.3)	0	0
Wisconsin	11 (36.7)	1 (33.3)	3 (10.7)
Age, y			
<18	10 (33.3)	0	7 (25.0)
>18	20 (66.7)	3 (100)	21 (75.0)
History of smallpox vaccination			
Yes	6 (20.0)	3 (100)	15 (53.6)
No	24 (80.0)	0	13 (46.4)
Setting in which exposure occurred*			
Home	18 (60.0)	1 (33.3)	17 (60.7)
Neighbor’s home	3 (10.0)	0	6 (21.4)
Pet store	2 (6.7)	1 (33.3)	1 (3.6)
Veterinary clinic	7 (23.3)	1 (33.3)	4 (14.3)
Exposure source†‡			
Prairie dog (PD)	30 (100)	3 (100)	28 (100)
PD and African animal	1 (3.3)	1 (33.3)	2 (7.1)
PD and other animal	1 (3.3)	0	2 (7.1)
Only other animal	0	0	0
Symptoms postexposure			
Fever	28§ (93.3)	2 (66.7)	4 (14.3)
Rash	30 (100)	1 (33.3)	7 (25.0)
Lymphadenopathy	20 (66.7)	1 (33.3)	4 (14.3)
Mouth sores	8 (26.7)	0	1 (3.6)
Conjunctivitis	4 (13.3)	0	0
Cough	17 (56.7)	0	2 (7.1)
Total	30	3	28

*Indicates principal setting for exposure. †Reported exposures pertain to animals with laboratory-confirmed monkeypox infections (*15*) or ones that had lesion-producing illnesses plus prior exposure to a confirmed infected animal. African animals included dormice, giant Gambian rats, jerboas, hedgehogs, striped mice, and pygmy mice. ‡Includes all reported exposures; categories not mutually exclusive. §Self-reported information for fever was indicated as unknown or was missing for 2 persons who had laboratory-confirmed monkeypox infections.

**Figure 1:**
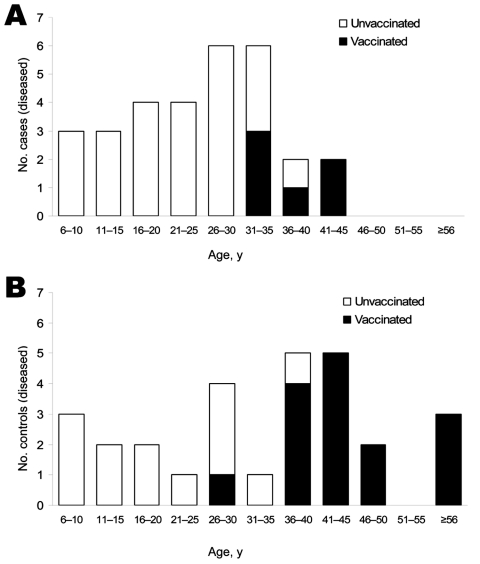
Age distribution of monkeypox virus–infected case-patients (A) and controls (B) and smallpox vaccination status. No study participants reported having received a smallpox vaccination within 25 years of August 2003.

Most persons enrolled were >18 years of age at the time of study initiation (Table 2). The mean age of case-patients at study enrollment (25.0 years) was significantly lower than that of controls (33.3 years; 2-sided p = 0.013, by Student *t* test); the median ages of the 2 groups were 27.0 and 36.5 years, respectively. Only 6 case-patients (20%), 3 IBND case-patients (100%), and 15 controls (53.6%) reported having had >1 smallpox vaccinations (Figure 1). All but one of the smallpox-vaccinated participants was >31 years of age at the time of the study, which is consistent with their having received routine vaccination before the 1972 abandonment of this practice in the United States (*18*). The only vaccinated study participant <31 years of age, a control, had been vaccinated as a child as a precaution for international travel.

### Characteristics of IBND Patients

Three persons classified as MPX IBND patients were adults (mean age 40.7 years, median age 38.0 years), and all reported having had >1 smallpox vaccination before 1972; 2 had visible vaccination scars. Symptoms of systemic illness (chills, sweats, myalgias) developed in all 3 patients in the 21 days after last known exposure to an MPX-infected prairie dog (Table 2), but classic MPX disease (1 reported papular rash without fever and 2 had fever without lesions or rash) did not develop. Two persons reported having touched infected prairie dogs (handled >1 infected animal), and the third had only indirect exposure to an infected animal (touched soiled bedding near an infected animal); none reported a bite or scratch from an ill prairie dog. The settings in which these persons were exposed differed for each person.

### Risk Factors for Acquiring MPX

Evaluation of associations between exposure variables and MPX disease outcomes with inclusion of unadjusted OR, adjusted OR (aOR, adjusted for history of smallpox vaccination), and 95% CI is shown in Table 3. Results relating to MPX disease as an outcome measure (i.e., that excluded the 3 IBND patients from the case group) are shown, unless results were qualitatively different when the 3 IBND patients were included. In those instances, results are reported for the diseased plus the 3 IBND patients.

**Table 3:** Smallpox vaccination status, demographic characteristics, and potential exposures to infected prairie dogs among case-patients and controls, monkeypox virus outbreak, United States, 2003*

Characteristic	Case-patients, no. (%)	Controls, no. (%)	OR (95% CI)	aOR (95% CI)
Smallpox vaccination				
Yes	6 (20.0)	15 (53.6)	0.2 (0.1–0.7)	
No	24 (80.0)	13 (46.7)		
Age, y†				
<18	10 (33.3)	7 (25.0)	1.5 (0.5–4.7)	0.6 (0.2–2.4)
>18	20 (66.7)	21 (75.0)		
Sex				
Female	17 (56.7)	16 (57.1)	1.0 (0.3–2.8)	0.9 (0.3–2.7)
Male	13 (43.3)	12 (42.9)		
Type of exposure to prairie dog(s)				
Animal as pet				
Yes	17 (56.7)	9 (32.1)	2.8 (0.9–8.1)	2.4 (0.8–7.4)
No	13 (43.3)	19 (67.9)		
Daily exposure while animal was ill				
Yes	23 (76.7)	13 (46.4)	3.8 (1.2–11.7)	4.0 (1.2–13.4)
No	7 (23.3)	15 (53.6)		
Touched rash or eye crusts				
Yes	18 (60.0)	11 (39.3)	2.3 (0.8–6.6)	2.2 (0.7–6.7)
No	12 (40.0)	17 (60.7)		
Scratched‡				
Yes	9 (30.0)	2 (7.1)	5.6 (1.1–28.6)	3.9 (0.7–21.1)
No	21 (70.0)	26 (92.9)		
Cleaned cage/touched bedding				
Yes	14 (46.7)	4 (14.3)	5.3 (1.5–18.9)	5.3 (1.4–20.7)
No	16 (53.3)	24 (85.7)		
Proximity, no touching§				
Yes	12 (40.0)	7 (25.0)	2.0 (0.6–6.2)	2.0 (0.6–6.5)
No	18 (60.0)	21 (75.0)		
Direct exposure¶				
Yes	23 (76.7)	13 (46.4)	3.8 (1.2–11.7)	4.0 (1.2–13.4)
No	7 (23.3)	15 (53.6)		

*OR, odds ratio; CI, confidence interval; aOR, adjusted OR. The aORs and 95% CIs were adjusted for history of (>1) smallpox vaccination at any time during case-patient’s life. †Routine immunization was stopped in the United States in 1972 (*18*), persons <33 years of age at the time of the outbreak were unlikely to have received prior smallpox vaccination. ‡Scratched with a break in the skin. §Came within 6 feet of the prairie dog but never touched it. ¶Direct exposure to an infected animal included having touched the animal or having received a bite or scratch from it sufficient to break the skin.

When not considering participant age or exposure history, a history of remote smallpox vaccination (>25 years previous) was protective against acquisition of MPX illness (OR 0.2, 95% CI 0.1–0.7). However, smallpox vaccination status was highly correlated with age (Spearman rank-order correlation r^2^ = 0.809, p<0.001), and its effect independent of age could not be assessed. Age group and sex did not differ between case-patients and controls.

Several types of direct exposure (touching or receiving a bite or scratch sufficient to break the skin) and indirect exposure (nontactile) with infected animals were associated with risk for MPX. Regarding direct exposures, having touched a sick animal was significantly associated with development of MPX (OR 3.8, 95% CI 1.2–11.7) regardless of smallpox vaccination status (aOR 4.0, 95% CI 1.2–13.4). Having received a scratch from an infected animal was significantly associated with disease but only when not adjusted for smallpox vaccination (OR 5.6, 95% CI 1.1–28.6; aOR 3.9, 95% CI 0.7–21.1). Among 9 persons who were scratched by an infected prairie dog, 1 had had prior smallpox vaccination. Only 1 MPX case-patient (a previously vaccinated person) reported a prairie dog bite; no controls reported having been bitten.

Among indirect exposures, having been near an infected animal (defined as having come within 6 feet of the animal without touching it) was not associated with development of MPX. Having cleaned the cage or touched used bedding of an infected animal was significantly associated with MPX development, both with and without adjustment for prior smallpox vaccination (OR 5.3, 95% CI 1.5–18.9; aOR 5.3, 95% CI 1.4–20.7). Also, regardless whether exposure was direct or indirect, having had daily exposure to the animal while it was ill was significantly associated with MPX developing (OR 3.8, 95% CI 1.2–11.7; aOR 4.0, 95% CI 1.2–13.4).

When MPX infection (n = 33, 30 patients and 3 IBND patients), rather than MPX disease, was chosen as the outcome measure, only direct (touch) exposure (OR 5.0, 95% CI 1.4–17.6; aOR 4.9, 95% CI 1.3–17.9) and having cleaned the cage or touched used bedding of an infected animal (OR 3.6, 95% CI 1.2–10.7; aOR 3.6, 95% CI 1.2–11.4) were significantly associated with infection. Again, regardless of age or exposure characteristics, reported history of prior, remote smallpox vaccination was protective against MPX infection (OR 0.3, 95% CI 0.1–0.9).

### Exposure Characteristics of Vaccinated and Unvaccinated Adults and Children

Because the study population was too small to support multivariate analysis, we were unable to evaluate associations between disease outcomes and individual exposures while controlling for other potentially contributing factors. Therefore, we evaluated types of exposures to prairie dogs among different study subgroups (smallpox-vaccinated persons vs. unvaccinated persons, and adults vs. children) to examine whether differences in proportions exposed could have biased observed associations between disease acquisition and previous smallpox vaccination or age.

**Figure 2:**
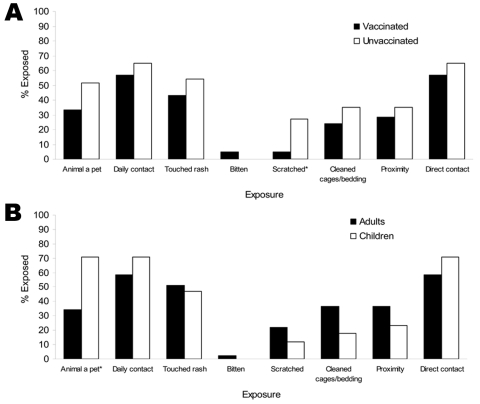
Characteristics of exposure to infected prairie dogs of A) vaccinated and unvaccinated monkeypox case-patients and controls and B) case-patients (adults and children) and controls. *Denotes statistically significant differences in exposure between groups.

The proportion of smallpox-vaccinated and unvaccinated study participants who reported various exposures to infected prairie dogs is shown in Figure 2, panel A. For all but 1 category of exposure, a smaller proportion of smallpox-vaccinated persons reported exposure than unvaccinated persons, which potentially introduced bias in interpreting lack of prior vaccination as a risk factor for disease. The exception, having been bitten by an infected animal, was reported by only 1 person, a smallpox-vaccinated person in whom relatively benign MPX developed. This person was not hospitalized and had <25 lesions. Unvaccinated persons had a higher frequency of scratches and breaks in the skin (10 [27.0%]) than did smallpox-vaccinated persons (1 [4.8%]; p = 0.044, by 2-tailed Fisher exact test). A similar evaluation of exposure characteristics in children and adults indicated that while certain exposures were more common in children than in adults (e.g., having had an infected animal as a pet, having had daily or direct exposure to an infected animal) (Figure 2, panel B), neither age category uniformly had higher proportionate exposure.

### Risk Factors for Vaccinated and Unvaccinated Participants

To evaluate potential differences in risks for MPX disease acquisition between smallpox-vaccinated and unvaccinated persons, case-patients and controls were stratified by vaccination status and exposures were examined. No exposure or demographic variables were associated with infection or disease status when participants were stratified by smallpox vaccination status. However, power to detect statistically significant associations was limited because of small numbers.

## Discussion

Although complexity of exposures varied among persons affected during the 2003 outbreak of MPX in the United States, prairie dogs were the common vehicle implicated in virus transmission to humans (*3*,*8*,*19*,*20*). The goal of this study was to identify host characteristics and specific types of exposure to infected animals that were associated with increased risk for infection or disease. Results demonstrated that several types of direct and indirect exposures to infected animals (including being scratched by an infected animal, handling one, or cleaning a soiled cage) were associated with increased risk of acquiring MPX. The observation that daily exposure to infected prairie dogs was associated principally with disease, and not merely with infection, suggests that increasing the intensity or duration of exposure to viral inoculum may increase the probability for overt illness.

Insights into potential transmission mechanisms by which handling or exposure to excretions or secretions of an infected animal could result in human infection come from pathology of MPXV infection in prairie dogs. Immunohistochemical evaluation of lesion and organ tissue obtained from prairie dogs that became ill during the US outbreak showed abundant viral antigen in surface epithelial cells at ulcerated sites on the tongue, conjunctiva, and throughout the lungs (*5*). Results of experimental infection studies of prairie dogs with MPX mirrored these observations (*16*), which suggests that respiratory, mucocutaneous, and transdermal routes of virus transmission are plausible, all of which are consistent with types of exposures implicated in this study.

Although a history of smallpox vaccination was associated with diminished risk for MPXV infection in this study, accurately interpreting this finding is difficult in the absence of consideration of age or exposure history. In human epidemiologic studies, age has been shown to affect susceptibility to MPX. Additionally, studies conducted in the 1980s in the Democratic Republic of the Congo (former Zaire) highlighted both age and smallpox vaccination status as key determinants of MPX acquisition in humans; unvaccinated children 5–15 years of age, those old enough for autonomous interaction with animals, had the highest rates of MPXV infection (*21*–*23*). In these studies, a prior history of smallpox vaccination within 3–19 years before MPXV exposure provided up to 85% protection against MPXV infection across various age classes. In our study, no study participants received smallpox vaccination within 25 years of MPX exposure, and the role of vaccination could not be evaluated independent of age because there were few unvaccinated older persons in the study population and no vaccinated children. Lack of adequate comparison groups needed to discriminate between age- and vaccination-related effects could emphasize potential protective benefits of remote smallpox vaccination more than older age at disease acquisition.

Also complicating interpretation of the role of vaccination in influencing susceptibility is our observation that vaccinated participants had fewer overall exposures to infected animals than did unvaccinated participants. Vaccinated persons had fewer invasive (scratch) exposures, which were associated with the highest case conversion rate (81.8%) among all measured exposures (except bite exposure, which was recorded only 1 time). Thus, discerning whether vaccinated participants benefited from having had fewer exposures to infected animals or from residual smallpox vaccine–derived immunity was difficult. The disproportionate enrollment of controls in different states (e.g., less for Wisconsin, where many exposures occurred in veterinary care settings), may have contributed to this effect. Because size and composition of the study population were insufficient to support multivariate modeling approaches for assessment of vaccination status, age, and exposure as independent risk factors, we were unable to clearly define a protective benefit of remote vaccination against acquisition of MPXV infection.

Although suggested in 1 report (*24*), systematically collected data from patients infected with MPXV of the West African genetic clade during the outbreak in the United States have not supported a role for remote smallpox vaccination in mitigation of MPX disease severity (*25*). In our study, mild subclinical MPXV infections occurred in 3 adult study participants, all of whom were previously vaccinated. Whether the diminished severity was a consequence of the manner of exposure, the age of the person, the person’s vaccination status, or some other factor is unknown. No common exposure was identified among these persons, and each reported symptoms of viral illness. All had evidence of a specific immunologic response after exposure to MPXV. Subclinical and asymptomatic MPXV infections have been suggested in both children and adults in other studies (*21*,*22*,*24*), but we provide evidence of mild, systemic, nonspecific symptoms in the context of an acute (IgM) antibody response. The epidemiologic role of such cases remains to be determined, but their identification here supports the need for investigation of persons with nonspecific symptoms during future MPX outbreak investigations.

The route of MPXV infection (bite, mucocutaneous) influences the time course and manifestations of illness (*10*). We have assessed overall risk for infection associated with types of exposures. It is arguable that behavioral interactions between prairie dogs and pet owners or veterinarians have no direct parallels with types of animal-human interactions that result in MPXV infections in African settings. However, results of this study highlight the role of direct physical contact and potential exposure to excretions and secretions of a sick animal (urine, feces, saliva). These results also provide insights into how natural infections might occur in Africa (e.g., not only during preparation of carcasses, as has traditionally been suggested, but also through exposure to animal nests or excreta). There are no smallpox vaccination programs ongoing in Africa, and smallpox vaccines currently licensed are not considered suitable for use in populations with high prevalences of immunocompromised persons (*26*,*27*). Behavioral interventions offer the best opportunity to prevent introduction of MPXV into human communities.

## References

[1] Ladnyj ID, Ziegler P, Kima E. A human infection caused by monkeypox virus in Basankusu Territory, Democratic Republic of the Congo. Bull World Health Organ. 1972;46:593–7. 4340218 PMC2480792

[2] von Magnus P, Andersen E, Petersen K, Birch-Andersen A. A pox-like disease in *Cynomolgus* monkeys. Acta Pathol Microbiol Scand. 1959;46:156–76.

[3] Centers for Disease Control and Prevention. Update: multistate outbreak of monkeypox—Illinois, Indiana, Kansas, Missouri, Ohio, and Wisconsin, 2003. MMWR Morb Mortal Wkly Rep. 2003;52:642–6. 12855947

[4] Arita I, Jezek Z, Khodakevich L, Ruti K. Human monkeypox: a newly emerged orthopoxvirus zoonosis in the tropical rain forests of Africa. Am J Trop Med Hyg. 1985;34:781–9. 2992305 10.4269/ajtmh.1985.34.781

[5] Guarner J, Johnson BJ, Paddock CD, Shieh WJ, Goldsmith CS, Reynolds MG, Monkeypox transmission and pathogenesis in prairie dogs. Emerg Infect Dis. 2004;10:426–31. 15109408 10.3201/eid1003.030878 PMC3322777

[6] Khodakevich L, Jezek Z, Kinzanzka K. Isolation of monkeypox virus from wild squirrel infected in nature. Lancet. 1986;1:98–9. 10.1016/S0140-6736(86)90748-8 2867342 PMC9631390

[7] Mutombo M, Arita I, Jezek Z. Human monkeypox transmitted by a chimpanzee in a tropical rain-forest area of Zaire. Lancet. 1983;1:735–7. 10.1016/S0140-6736(83)92027-5 6132084 PMC9534202

[8] Reed KD, Melski JW, Graham MB, Regnery RL, Sotir MJ, Wegner MV, The detection of monkeypox in humans in the Western Hemisphere. N Engl J Med. 2004;350:342–50. 10.1056/NEJMoa032299 14736926

[9] Fleischauer AT, Kile JC, Davidson M, Fischer M, Karem KL, Teclaw R, Evaluation of human-to-human transmission of monkeypox from infected patients to health care workers. Clin Infect Dis. 2005;40:689–94. 10.1086/427805 15714414

[10] Reynolds MG, Yorita KL, Kuehnert MJ, Davidson WB, Huhn GD, Holman RC, Clinical manifestations of human monkeypox influenced by route of infection. J Infect Dis. 2006;194:773–80. 10.1086/505880 16941343

[11] Likos AM, Sammons SA, Olson VA, Frace AM, Li Y, Olsen-Rasmussen M, A tale of two clades: monkeypox viruses. J Gen Virol. 2005;86:2661–72. 10.1099/vir.0.81215-0 16186219

[12] Karem KL, Reynolds M, Braden Z, Lou G, Bernard N, Patton J, Characterization of acute-phase humoral immunity to monkeypox: use of immunoglobulin M enzyme-linked immunosorbent assay for detection of monkeypox infection during the 2003 North American outbreak. Clin Diagn Lab Immunol. 2005;12:867–72. 16002637 10.1128/CDLI.12.7.867-872.2005 PMC1182207

[13] Olson VA, Laue T, Laker MT, Babkin IV, Drosten C, Shchelkunov SN, Real-time PCR system for detection of orthopoxviruses and simultaneous identification of smallpox virus. J Clin Microbiol. 2004;42:1940–6. 10.1128/JCM.42.5.1940-1946.2004 15131152 PMC404623

[14] Centers for Disease Control and Prevention. Update: multistate outbreak of monkeypox—Illinois, Indiana, Kansas, Missouri, Ohio, and Wisconsin, 2003. MMWR Morb Mortal Wkly Rep. 2003;52:561–4. 12816106

[15] Hutson CL, Lee KN, Abel J, Carroll DS, Montgomery JM, Olson VA, Monkeypox zoonotic associations: insights from laboratory evaluation of animals associated with the multi-state US outbreak. Am J Trop Med Hyg. 2007;76:757–68. 17426184

[16] Xiao SY, Sbrana E, Watts DM, Siirin M, da Rosa AP, Tesh RB. Experimental infection of prairie dogs with monkeypox virus. Emerg Infect Dis. 2005;11:539–45. 15829191 10.3201/eid1104.040907 PMC3320329

[17] Kleinbaum DG, Klein M. Logistic regression, a self-learning text, 2nd ed. New York: Springer-Verlag; 2002.

[18] Advisory Committee for Immunization Practices (ACIP). Smallpox vaccine. MMWR Morb Mortal Wkly Rep. 1980;29:417–20.

[19] Anderson MG, Frenkel LD, Homann S, Guffey J. A case of severe monkeypox virus disease in an American child: emerging infections and changing professional values. Pediatr Infect Dis J. 2003;22:1093–6. 10.1097/01.inf.0000101821.61387.a5 14688573

[20] Sejvar JJ, Chowdary Y, Schomogyi M, Stevens J, Patel J, Karem K, Human monkeypox infection: a family cluster in the midwestern United States. J Infect Dis. 2004;190:1833–40. 10.1086/425039 15499541

[21] Jezek Z, Marennikova SS, Mutumbo M, Nakano JH, Paluku KM, Szczeniowski M. Human monkeypox: a study of 2,510 contacts of 214 patients. J Infect Dis. 1986;154:551–5. 3018091 10.1093/infdis/154.4.551

[22] Jezek Z, Nakano JH, Arita I, Mutombo M, Szczeniowski M, Dunn C. Serological survey for human monkeypox infections in a selected population in Zaire. J Trop Med Hyg. 1987;90:31–8. 3029395

[23] Jezek Z, Grab B, Paluku KM, Szczeniowski MV. Human monkeypox: disease pattern, incidence and attack rates in a rural area of northern Zaire. Trop Geogr Med. 1988;40:73–83. 2841783

[24] Hammarlund E, Lewis MW, Carter SV, Amanna I, Hansen SG, Strelow LI, Multiple diagnostic techniques identify previously vaccinated individuals with protective immunity against monkeypox. Nat Med. 2005;11:1005–11. 16086024 10.1038/nm1273

[25] Huhn GD, Bauer AM, Yorita K, Graham MB, Sejvar J, Likos A, Clinical characteristics of human monkeypox, and risk factors for severe disease. Clin Infect Dis. 2005;41:1742–51. 10.1086/498115 16288398

[26] Edghill-Smith Y, Venzon D, Karpova T, McNally J, Nacsa J, Tsai WP, Modeling a safer smallpox vaccination regimen, for human immunodeficiency virus type 1–infected patients, in immunocompromised macaques. J Infect Dis. 2003;188:1181–91. 10.1086/378518 14551889

[27] Edghill-Smith Y, Bray M, Whitehouse CA, Miller D, Mucker E, Manischewitz J, Smallpox vaccine does not protect macaques with AIDS from a lethal monkeypox virus challenge. J Infect Dis. 2005;191:372–81. 10.1086/427265 15633096

